# In vivo assessment of GABAergic inhibition and glutamate facilitation in treatment-resistant schizophrenia: a TMS study integrating clinical, cognitive, and neurophysiological evaluations

**DOI:** 10.1038/s41537-025-00634-w

**Published:** 2025-06-19

**Authors:** Annarita Barone, Gianmaria Senerchia, Giuseppe De Simone, Marco Manzo, Mariateresa Ciccarelli, Stefano Tozza, Valentina Virginia Iuzzolino, Myriam Spisto, Raffaele Dubbioso, Felice Iasevoli, Rosa Iodice, Andrea de Bartolomeis

**Affiliations:** 1 Section of Psychiatry, Laboratory of Molecular and Translational Psychiatry, Unit of Treatment-Resistant Psychiatric Disorders, Department of Neuroscience, Reproductive Sciences and Dentistry, University of Naples “Federico II”, Naples, Italy; 2 Neurology Unit, Department of Neuroscience, Reproductive Sciences and Dentistry, University of Naples “Federico II”, Naples, Italy; 3 Department of Psychology, University of Campania Luigi Vanvitelli, Naples, Italy

**Keywords:** Neural circuits, Schizophrenia

## Abstract

Treatment-resistant schizophrenia (TRS) affects approximately one-third of individuals with schizophrenia, posing significant challenges for clinical management. Clozapine treatment is often delayed, underscoring the urgent need for an early potential signature of TRS. To date, specific alterations in cortical excitability and plasticity underlying TRS remain unexplored. We evaluated cortical excitability and plasticity in 30 patients with schizophrenia (15 TRS, 15 non-TRS) and 21 controls using transcranial magnetic stimulation (TMS). Measures included motor thresholds and protocols probing GABAergic inhibition and glutamatergic facilitatory activity, the excitation index (EI) in the primary motor cortex (M1), and long-term potentiation (LTP)-like plasticity using intermittent theta burst stimulation (iTBS). Clinical severity and cognitive performance were evaluated using the Positive and Negative Syndrome Scale (PANSS) and the Brief Assessment of Cognition in Schizophrenia (BACS). TRS patients exhibited significantly higher active motor thresholds (*p* = 0.015) and impaired short-interval intracortical inhibition (SICI) (*p* = 0.001) vs healthy controls, reflecting GABAergic dysfunction. EI was elevated in TRS *v*s non-TRS patients (*p* = 0.034) and controls (*p* = 0.002), indicating pronounced cortical hyperexcitability. Both TRS (*p* = 0.008) and non-TRS patients (*p* = 0.033) showed reduced plasticity following iTBS compared to controls, with no TRS vs non-TRS difference. SICI deficits significantly correlated with negative (*r* = 0.524, *p*_adj_ = 0.03) and autistic (*r* = 0.517, *p*_adj_ = 0.03) symptom severity as assessed by the PANSS negative score and Positive and Negative Syndrome Scale Autism Severity Score (PAUSS). Our findings point to a neurophysiological continuum in schizophrenia, with TRS patients demonstrating the most pronounced cortical hyperexcitability and impaired plasticity, and non-TRS patients showing intermediate deficits.

## Introduction

Schizophrenia is a severe psychiatric disorder affecting approximately 1% of the global population, characterized by profound impairments in cognition, perception, and behavior. Approximately one-third of patients fail to achieve an objective significant improvement after two trials with different antipsychotics at adequate doses and for a treatment duration of 6 weeks each^1^. These patients are considered treatment-resistant schizophrenia (TRS) patients. TRS is associated with significant reduction both in quality of life and functioning^2^ and imposes a substantial economic burden on patients and society^1^.

For TRS, clozapine is the only pharmacological treatment specifically approved, with evidence supporting a greater effectiveness when introduced early^3^. However, clozapine introduction in therapy is frequently delayed, resulting in prolonged exposure to ineffective medications. Efforts to identify reliable biological correlates for earlier diagnosis and targeted interventions remain crucial to advancing patient outcomes^4^.

Evidence suggests that TRS may have distinct biological underpinnings compared to treatment-responsive schizophrenia. Dysregulation of glutamatergic activity, mainly within the prefrontal cortex, is thought to drive persistent positive symptoms and cognitive impairments in TRS^5–7^ in line with the pivotal role of glutamate in synaptic plasticity both at the micro domain (dendritic spine) and macro domain (cortical–subcortical integration) level^8^. Additionally, the disruption of cortical excitatory–inhibitory balance, largely due to impaired function of GABAergic interneurons may contribute to the persistence of symptoms despite adequate dopaminergic blockade^9–11^. However, important gaps remain: which specific circuits are most disrupted in TRS, and how do they map onto the symptom profile? If GABAergic alterations play a role, which GABA receptor subunits are most critically altered in TRS? How do *N*-methyl-d-aspartate (NMDA)-dependent plasticity deficits differ between responsive and non-responsive patients?

Understanding these pathways more deeply could refine treatment strategies and facilitate the development of novel therapies, particularly for patients unresponsive to clozapine^1,12^.

Transcranial magnetic stimulation (TMS) has emerged as a safe^13^ and valuable neurophysiological tool for studying alterations in cortical circuitry across psychiatric and neurological conditions, including schizophrenia^14–17^. While TMS studies in schizophrenia have produced heterogeneous results, the most consistent finding is a reduction in short-interval intracortical inhibition (SICI)^16^. Findings for other measures, such as motor thresholds, intracortical facilitation (ICF), long-interval intracortical inhibition (LICI), and short-latency afferent inhibition (SAI), have been more variable. Several studies have investigated synaptic plasticity in schizophrenia using non-invasive brain stimulation protocols, reporting consistent disruptions in mechanisms underlying long-term potentiation (LTP) and long-term depression (LTD)^18^. Of note, one study employing a theta burst stimulation (TBS) protocol expected to induce LTP-like effects reported a paradoxical inhibitory response^19^.

The present study aims to identify neurophysiological signatures that may differentiate TRS from treatment-responsive schizophrenia. We hypothesize that TRS may significantly differ in TMS-based measures—including intracortical inhibition/facilitation, cortical plasticity, and excitation/inhibition balance—reflecting either distinct patterns of cortical circuit dysfunction or, at least, a progressive gradient of impairment across the three groups: healthy subjects, treatment-responsive, and TRS patients. A deeper understanding of these conditions could inform the development of more personalized and effective therapeutic approaches.

## Patients and methods

### Study participants and ethical approval

**Fig. 1:**
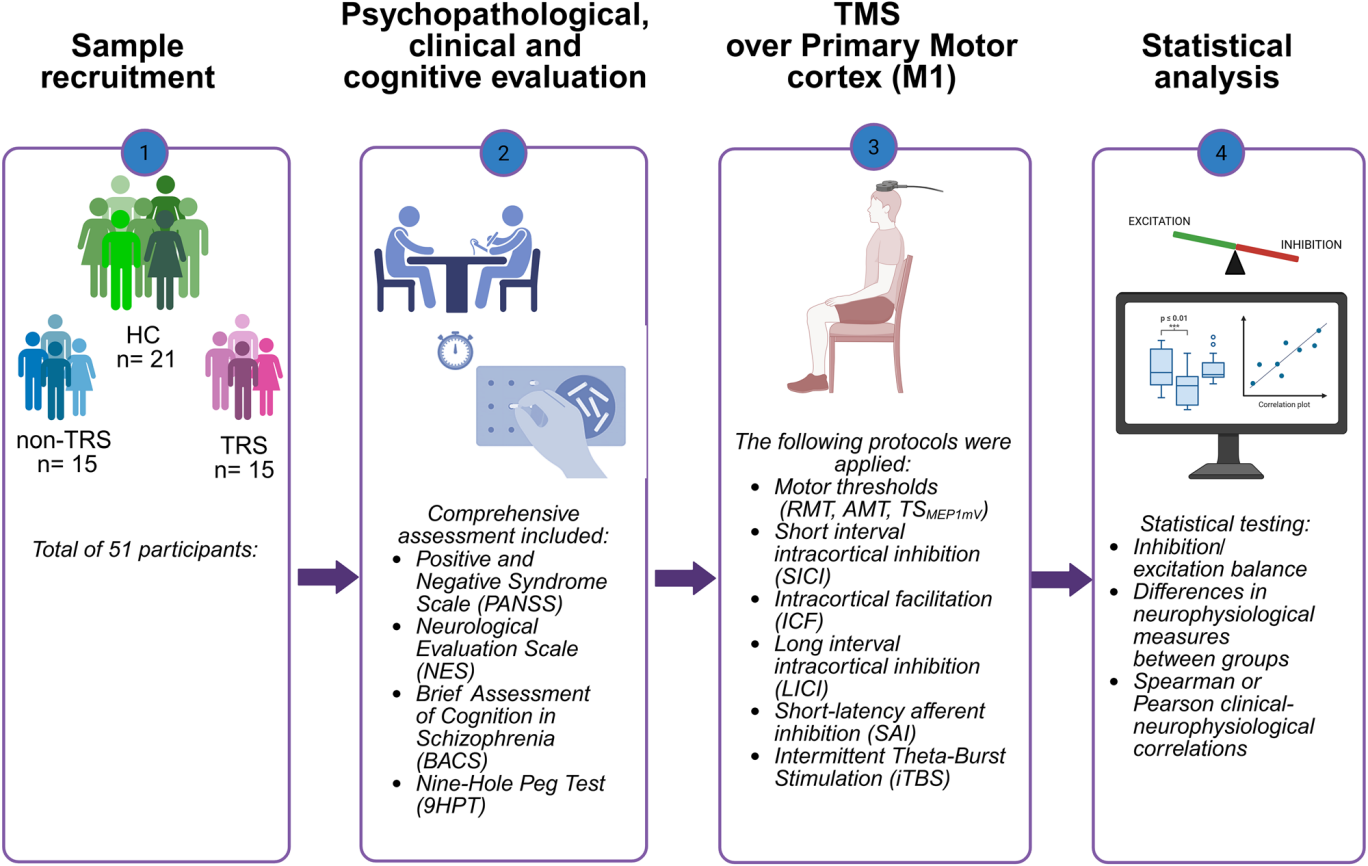
Schematic overview of the experimental study.

The study was conducted at the Outpatient Unit for Treatment Resistant Psychosis, Section of Psychiatry and jointly at the Neurophysiology Unit, Section of Neurology, both within the Department of Neuroscience, University “Federico II” of Naples, from November 2023 to September 2024. We enrolled 30 patients diagnosed with schizophrenia and 21 healthy controls (see Fig. 1); for inclusion and exclusion criteria, see Supplementary Material (Supplementary Tables 1 and 2). Participants were consecutively recruited to ensure representativeness and avoid selection bias. Statistical analyses confirmed no significant differences between groups in key demographic and clinical characteristics (age, age of onset, sex, disease duration, chlorpromazine and diazepam-equivalent doses), minimizing confounding effects. All research procedures adhered to the principles of the Declaration of Helsinki (1975, as revised in 2008) and received approval from the Ethical Committee of the University of Naples Federico II (protocol n. 65/2023). Written informed consent was obtained from all participants prior to their enrollment.

### Clinical evaluation

All consecutive patients who met the eligibility criteria were screened by two experienced psychiatrists to confirm the diagnosis according to DSM-5 criteria^20^. The following demographic and clinical data were gathered: age, sex, years of education, age at psychosis onset, duration of illness, number of hospitalizations, and current doses of medications expressed in chlorpromazine and diazepam equivalents (details of benzodiazepine type, daily dose range, and diazepam equivalents are presented in Supplementary Table 3)^21^. Patients were divided into TRS (*n* = 15) and non-TRS (*n* = 15) according to the American Psychiatric Association algorithm, as refined by the Treatment Response and Resistance in Psychosis Working Group consensus guidelines^22^. Hence, patients were classified as TRS if they had completed two adequate antipsychotic trials (each ≥6 weeks at ≥600 mg chlorpromazine equivalents) with a Positive and Negative Syndrome Scale (PANSS) total score reduction ≤20% (detailed TRS criteria are reported in Supplementary Table 4)^23^. If both the historical and current clinical severity criteria were satisfied, a novel antipsychotic agent was tested prospectively at an adequate dose and duration to confirm the diagnosis of TRS.

The comprehensive clinical assessment included validated scales: PANSS^24^ to assess symptom severity, applying the five-factor model that includes positive, negative, disorganization, excitement, and emotional distress factors^25^. Additionally, the Positive and Negative Syndrome Scale Autism Severity Score (PAUSS) was computed to recognize autistic symptoms^26^. Since the present study investigates excitatory–inhibitory balance, assessing autism spectrum features dimensionally (via PAUSS) may allow us to account for this neurodevelopmental dimension, which may contribute to the heterogeneity of clinical and neurochemical profiles, especially in TRS^27^. The Neurological Evaluation Scale (NES)^28^ was employed to evaluate neurological soft signs. Cognitive performance was evaluated across several domains using the Brief Assessment of Cognition in Schizophrenia: verbal memory was evaluated with the list learning task; working memory through the digit sequencing task; verbal fluency via the category instances task; motor coordination with the token motor task; processing speed using the symbol coding task; and problem-solving with the Tower of London task. Individual task scores were transformed into *z*-scores based on normative data to compute a composite cognitive index^29^.

### Hand dexterity task

Hand dexterity was assessed using the Nine-Hole Peg Test (9HPT). Both hands were tested, with three consecutive trials for the dominant hand and three consecutive trials for the nondominant hand. The time required to complete the task was recorded for each trial, and the average time and coefficient of variation were calculated for each hand^30,31^.

### Electromyographic recording and focal TMS

Participants were seated comfortably with their hands resting on a cushion for complete relaxation. Motor evoked potentials (MEPs) were recorded via electromyography (EMG) from the dominant first dorsal interosseous (FDI) muscle using Ag–AgCl surface electrodes in a belly-tendon montage. EMG signals were amplified, bandpass filtered (20Hz–3 kHz), digitized at 5 kHz, and stored for offline analysis. Trials with involuntary EMG activity exceeding 50 μV within 500 ms before MEPs were excluded.

Focal TMS was delivered using a figure-of-eight coil (70 mm diameter) positioned tangentially to the skull at a 45° angle to the sagittal plane (posterior-to-anterior current direction), connected to a MagPro X100 stimulator (Medtronic, Denmark). The optimal scalp position for eliciting maximal MEPs in the contralateral FDI (hotspot) was marked with a soft-tip pen. Peak-to-peak MEP amplitudes were measured and averaged^32^.

### TMS protocols exploring cortical inhibitory and facilitatory networks

The resting motor threshold (RMT) was defined as the minimum stimulator intensity producing MEP ≥ 50μV in 5 of 10 consecutive trials. Active motor threshold (AMT) was measured during mild contraction (~20% of maximal contraction) as the lowest intensity evoking MEPs ≥200 μV in 5 of 10 consecutive trials. MEP1mV was the lowest intensity eliciting 1 mV MEP in 5 of 10 consecutive trials. SICI and ICF were tested with a conditioning stimulus at 90% AMT, delivered before the test stimulus (TS). The unconditioned TS was adjusted to MEP1mV. SICI was assessed at interstimulus intervals (ISIs) of 2 and 3 ms, while ICF was tested at ISIs of 10 and 15 ms^33,34^.

The EI was calculated to provide a quantitative measure of the balance between ICF and SICI within the motor cortex. To compute the EI, the average of normalized SICI and ICF values was obtained across the different ISIs. The EI was then derived using the following formula: EI = ICF/(ICF − SICI), where ICF and SICI represent the mean facilitation and inhibition levels, respectively. This index reflects the relative contribution of excitatory and inhibitory circuits to the overall cortical excitability within M1. Higher EI values may reflect either an increase in ICF or a reduction in GABA-A-mediated inhibition, or both. Conversely, lower EI values suggest stronger inhibitory activity or reduced facilitation^35^.

Finally, SAI was analyzed at different ISIs based on the individual N20 wave latency. ISIs ranged from 0 to 4 ms after N20 latency, in steps of 2 ms. Data of patients and controls, obtained at the ISIs 0, 2, and 4, were analyzed and averaged to obtain a grand mean of SAI^36,37^. The median nerve was stimulated at the wrist through bipolar surface electrodes (cathode proximal, rectangular pulse of 0.2 ms duration). Stimulus intensity was adjusted to produce a slight thumb twitch (120% motor threshold). The intensity of TS was set to MEP1mV.

For all paired-pulse paradigms, 10 trials were collected for each condition and randomly intermixed with 10 trials of TS alone (0.2 ± 10% Hz). In addition, the mean peak-to-peak amplitude of the conditioned MEP at each ISI was represented as a percentage of the mean peak-to-peak amplitude size of the unconditioned test pulse in that block.

### Assessment of cortical plasticity after intermittent TBS

We applied iTBS using the paradigm introduced by Huang^38^. It consisted of bursts of 3 pulses at high frequency, 50 Hz, repeated at intervals of 200 ms, delivered in short trains lasting 2 s, with an 8-s pause between consecutive trains, for a total of 600 pulses. The stimulation intensity for iTBS was set at 80% AMT. To assess corticospinal excitability before iTBS, single MEPs were recorded using a stimulus intensity adjusted to produce MEP amplitude of approximately 1 mV in the relaxed FDI muscle. For each subject, 10 MEPs were acquired, and the peak-to-peak amplitudes were measured to determine the mean amplitude.

After the interventions, corticospinal excitability changes were tracked by collecting 10 MEP responses every 2 min following the intervention for up to 30 min (15 blocks, starting with 2 min of rest, followed by 1-min measurement and 1-min rest intervals). We decided to adopt a high temporal resolution of corticospinal excitability assessment after iTBS for a better evaluation of different patterns of motor cortex plasticity across the groups over time^35,39,40^.

### Statistical analysis

Data were analyzed using IBM SPSS Statistics v.29.0 for Windows (IBM, Armonk, NY). The normality of data distribution was verified using the Kolmogorov–Smirnov test. One-way analysis of variance (ANOVA) was used to compare age, motor thresholds, and EI across the three groups: TRS patients, non-TRS patients, and healthy controls.

The effects of SICI, ICF, LICI, and SAI were analyzed using separate two-way mixed-model ANOVAs, with “ISI” as the within-subject factor and “group” (TRS, non-TRS, and healthy controls) as the between-subject factor. For iTBS, a two-way mixed-model ANOVA was performed on MEP amplitude, expressed as a percentage change from baseline, with “time” as the within-subject factor and “group” as the between-subject factor. If a significant main effect was detected, post hoc analyses were performed using the least significant difference correction for multiple comparisons. The Greenhouse–Geisser correction was applied to adjust for nonsphericity where necessary. Moreover, to test whether group effects on outcome variables were independent of potential pharmacological confounders, an analysis of covariance (ANCOVA) was conducted to evaluate group differences in neurophysiological measures while controlling for medication dosage. Group (TRS vs non-TRS) was included as a fixed factor, and both diazepam-equivalent dose and chlorpromazine-equivalent dose were entered as covariates.

Correlations between clinical scores (e.g., symptom severity, cognitive performance) and key neurophysiological parameters were evaluated using Pearson’s correlation coefficient for normally distributed variables and Spearman’s rank correlation coefficient for non-normally distributed variables. In accordance with the Bonferroni correction method, *p* values were adjusted by multiplying the original *p* value by the number of comparisons performed (i.e., *p*_adj_ = *p* × *n*, where *n* is the total number of statistical tests). This correction was consistently applied to minimize the risk of type I errors resulting from multiple comparisons. Statistical significance was set at *p* < 0.05, and all data are reported as mean ± standard deviation unless otherwise specified.

## Results

### Demographic and clinical findings

The three groups did not differ significantly in terms of age and sex. However, patients were slower than healthy controls in performing the 9HPT. The mean completion time for the dominant hand was 18.66 s (interquartile range (IQR) 17.58–19.42) in healthy controls, compared to 26.73 s (IQR 22.86–31.95) in non-TRS patients and 27.73 s (IQR 22.68–34.59) for TRS patients (*p* < 0.001). For the non-dominant hand, healthy controls had a mean time of 19.66 s (IQR 18.24–20.57), while non-TRS and TRS patients had times of 27.70 s (IQR 22.86–31.95) and 28.25 s (IQR 24.20–32.93), respectively (*p* < 0.001) (Table 1). There was no significant difference in the coefficient of inter-trial variability for either the dominant or non-dominant hand between the groups (Table 1). Additionally, there were no significant differences between resistant and responsive patients regarding equivalent doses of chlorpromazine or diazepam (Table 1). While NES total scores did not show a significant difference between TRS and non-TRS, the PANSS total scores were significantly higher in TRS (88.93 [IQR: 70.00–104.00]) compared to non-TRS (67.13 [IQR: 54.00–71.00]; *p* = 0.007) (Table 1).

**Table 1:** Comparison of demographical and clinical variables.

Variable	HC	Non-TRS	TRS	*p* value
Sample size (*n*)	21	15	15	
Sex (M/F)	6/15	6/9	7/8	0.524
Age (years)	42.00 (34.00–50.00)	43.60 (36.00–54.50)	41.20 (34.50–47.50)	0.899
Dominant hand 9HPT mean time (s)	18.66 (17.58–19.42)*	26.73 (21.37–30.72)	27.73 (22.68–34.59)	**<0.001**
Non-dominant hand 9HPT mean time (s)	19.66 (18.24–20.57)*	27.70 (22.86–31.95)	28.25 (24.20–32.93)	**<0.001**
Dominant hand coefficient of inter-trial variability (%)	6.78 (3.10–10.34)	6.28 (3.29–9.69)	7.32 (3.13–11.74)	0.803
Non-dominant hand coefficient of inter-trial variability (%)	4.97 (2.09–7.83)	6.33 (3.21–4.90)	4.98 (3.29–6.99)	0.638
Age of onset (years)	–	28.00 (21.00–30.50)	22.40 (20.00–25.00)	0.261
Disease duration (years)	–	15.60 (8.50–21.00)	18.80 (13.00–23.50)	0.319
Chlorpromazine equivalent	–	364.73 (213.00–503.00)	501.33 (387.50–592.50)	0.067
Diazepam equivalent	–	3.23 (0.00–0.00)	7.77 (0.00–9.00)	0.064
PANSS total score	–	67.13 (54.00–71.00)	88.93 (70.00–104.00)	**0.007**
BACS *z*-score		−2.79 (−4.00–1.45)	−2.86 (−4.60–1.30)	0.965
NES total score	–	13.28 (6.00–19.00)	16.07 (9.00–21.00)	0.241

Note: Values are expressed as mean (interquartile range) or as frequencies and percentage. Comparisons between three groups were performed by means of non-parametric Kruskal–Wallis test; comparisons between two groups were performed by means of non-parametric Mann–Whitney *U*-test. Frequencies were compared by means of chi-square test. Values in bold type indicate significance *p* < 0.05.

*HC Healthy controls, 9HPT* Nine-Hole Peg Test, *PANSS* Positive and Negative Syndrome Scale, *BACS* Brief Assessment of Cognition in Schizophrenia, *NES* Neurological Evaluation Scale.

*Significantly different from the other two groups (*p* < 0.05).

### Motor thresholds

**Fig. 2:**
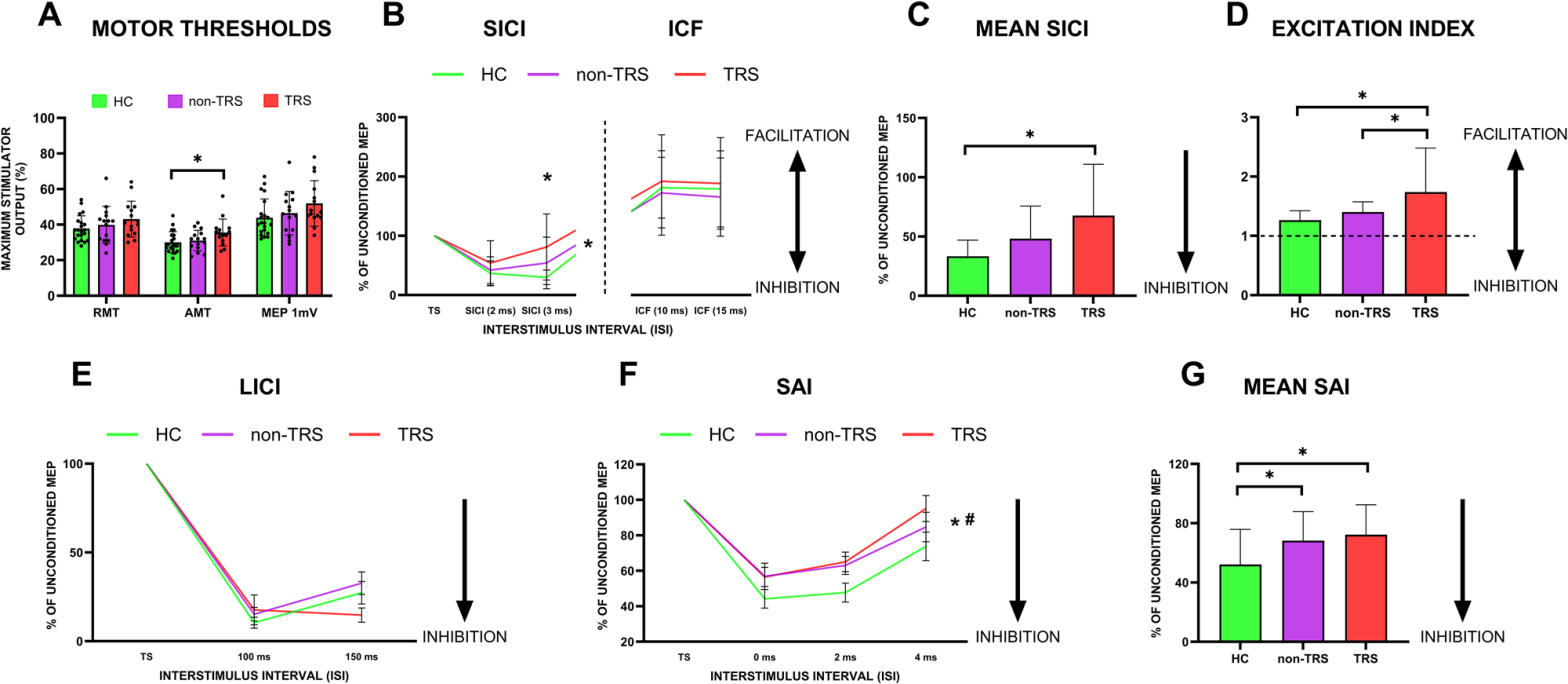
Cortical excitability and motor thresholds across TRS patients, non-TRS, and healthy controls. AMT was found significantly elevated in TRS patients compared to healthy controls, as shown in (**A**), while RMT and MEP1mV values did not differ significantly among the groups. The balance between short-interval intracortical inhibition SICI and ICF circuits has been presented in (**B**), where TRS demonstrate a reduction in SICI compared to controls (asterisk on the right side of the graph*)*, reflecting impaired inhibitory circuits, with a significant difference observed at 3 ms (asterisk above the bars). Mean SICI values (**C**), expressed as a percentage of the TS, were higher in TRS than in controls, further indicating reduced inhibition. The excitation index, which reflects the balance between inhibitory and excitatory circuit activity, is significantly increased in TRS patients compared to both healthy controls and non-TRS patients, suggesting a shift toward cortical excitation (**D**). **E** presents LICI data across interstimulus intervals, showing no significant differences among the groups. **F** presents SAI data, where TRS and non-TRS patients show a reduced inhibition compared to healthy controls. *Statistically significant for healthy controls *vs* TRS. ^#^Statistically significant for healthy controls vs non-TRS. **G** presents mean SAI data, where TRS and non-TRS patients show a reduced inhibition compared to healthy controls.

A one-way ANOVA comparing motor thresholds across the three groups revealed a significant difference only for AMT (*F*_2,50_ = 3.405, *p* = 0.041), but not for RMT (*F*_2,50_ = 1.531, *p* = 0.227) or MEP1mV (*F*_2,50_ = 2.076, *p* = 0.137). Post hoc analysis confirmed that the significant difference in AMT was present only between the TRS group and healthy controls (TRS vs healthy controls *p* = 0.015), with TRS patients showing elevated AMT values compared to healthy participants (Fig. 2A). A comprehensive overview of the neurophysiological data is provided in Table 2.

**Table 2:** Comparison of neurophysiological parameters across groups.

	HC	Non-TRS	TRS	*p* value
RMT Mean ± SD (IQR) (% of MSO)	37.76 ± 6.63 (31.50–41.50)	39.86 ± 10.64 (31.00–45.00)	43.13 ± 10.05 (35.00–50.00)	0.227
AMT Mean ± SD (IQR) (% of MSO)	30.04 ± 5.01 (25.00–34.00)	31.06 ± 5.91 (27.00–35.00)	35.53 ± 7.55 (31.00–38.00)	**0.041**
MEP1mV Mean ± SD (IQR) (% of MSO)	43.90 ± 9.64 (35.50–49.50)	46.46 ± 12.65 (37.00–54.00)	51.93 ± 12.80 (44.00–60.00)	0.137
*I* (mean SICI) Mean ± SD (IQR) (% of unconditioned MEP)	33.25 ± 13.63 (21.87–44.95)	48.19 ± 27.55 (25.40–64.34)	67.65 ± 43.31 (24.36–80.82)	**0.004**
*E* (mean ICF) Mean ± SD (IQR) (% of unconditioned MEP)	180.12 ± 46.90 (133.11–218.29)	168.87 ± 63.26 (114.53–194.88)	190.08 ± 76.27 (130.43–242.14)	0.661
EI Mean (IQR)	1.26 ± 0.18 (1.12–1.41)	1.40 ± 0.19 (1.27–1.55)	1.74 ± 0.74 (1.24–1.88)	**0.006**
Mean LICI Mean ± SD (IQR) (% of unconditioned MEP)	18.85 ± 19.93 (4.96–35.02)	23.93 ± 16.44 (4.97–37.78)	16.16 ± 22.65 (3.26–22.92)	0.523
Mean SAI Mean ± SD (IQR) (% of unconditioned MEP)	52.17 ± 28.23 (32.78–73.40)	68.21 ± 20.19 (55.81–86.62)	72.26 ± 20.22 (67.44–87.27)	**0.017**
Mean LTP Mean ± SD (IQR) (% of unconditioned MEP)	160.26 ± 70.83 (118.83–199.16)	131.98 ± 64.73 (86.06–159.26)	116.57 ± 29.54 (96.64–133.24)	**0.040**

Significance threshold set at *p* < 0.05. Bold values denote statistically significant results.

*HC healthy controls, RMT* resting motor threshold, *AMT* active motor threshold, MEP1mV threshold to obtain 1 mV amplitude MEP, *I* mean inhibition, *SICI* short-interval intracortical inhibition, *E* mean facilitation, *ICF* intracortical facilitation, *EI* excitation index, *LICI* long-interval intracortical inhibition, *SAI* short-latency afferent inhibition, *LTP* long-term plasticity.

### Intracortical inhibition and facilitation

SICI was calculated as the ratio of the conditioned to unconditioned MEP amplitude, with higher values indicating reduced GABA-A-mediated intracortical inhibition. A mixed-model ANOVA revealed a significant main effect of group (*F*_2,48_ = 6.122, *p* = 0.004), indicating that SICI modulation differs across TRS, non-TRS, and healthy controls. Post-hoc comparisons showed that TRS exhibited an altered modulation of intracortical inhibitory circuits, as tested by SICI, compared to healthy controls (*p* = 0.001), but there was no significant difference between TRS and non-TRS patients (Fig. 2B, C). As expected, we also observed a main effect of ISI (*F*_1,48_ = 4.879, *p* = 0.032), indicating significant modulation across different ISIs (Fig. 2B). Additionally, the interaction between ISI and group was significant (*F*_2,48_ = 4.468, *p* = 0.017). Post hoc pairwise comparisons revealed that this significant interaction was primarily due to a difference at the 3 ms ISI between controls and TRS patients, with TRS patients exhibiting significantly altered SICI modulation compared to healthy controls (*p* = 0.001). No significant differences were observed between non-TRS patients and the other groups at this ISI.

To further explore the GABA-A-ergic alteration of circuits in M1, we conducted a one-way ANOVA comparing mean SICI across the three groups, which revealed a significant group effect (*F*_2,48_ = 6.122, *p* = 0.004). Post-hoc analyses indicated that TRS patients (67.65 ± 43.31) exhibited reduced intracortical inhibition compared to healthy controls (33.25 ± 13.63, *p* = 0.001), but did not significantly differ from non-TRS patients (48.19 ± 27.55, *p* = 0.135) (Fig. 2C).

Additionally, non-TRS patients also showed a trend toward higher mean SICI values compared to healthy controls (*p* = 0.073). These findings suggest a notable alteration in inhibitory modulation within the motor cortex in TRS patients.

For ICF, a mixed-model ANOVA revealed no significant main effect of ISI (*F*_1,48_ = 0.427, *p* = 0.516) and no significant ISI × group interaction (*F*_2,48_ = 0.056, *p* = 0.945), indicating similar ICF modulation patterns across the three groups (TRS, non-TRS, and healthy controls), regardless of ISI. Additionally, there was no significant main effect of group (*F*_2,48_ = 0.417, *p* = 0.661), suggesting that overall ICF responses did not significantly differ between the groups.

Regarding the balance between inhibitory and facilitatory circuits in M1, we applied one-way ANOVA comparing the EI across the three groups, revealing a significant group effect (*F*_2,48_ = 5.608, *p* = 0.006). Post hoc analyses indicated significant differences between TRS patients (1.739 ± 0.741) and both non-TRS patients (1.401 ± 0.195, *p* = 0.034) and healthy controls (1.263 ± 0.185, *p* = 0.002), with TRS patients showing a higher EI compared to the other two groups (Fig. 2D). This suggests a shift in the balance toward net motor cortex hyperexcitability in TRS patients, potentially reflecting reduced inhibitory modulation.

To further explore inhibitory circuits, LICI was used. A mixed-model ANOVA revealed no significant main effect of group (*F*_2,48_ = 0.657, *p* = 0.523) (Fig. 2E), but a significant main effect of ISI (*F*_1,48_ = 8.092, *p* = 0.007), indicating a different modulation of the inhibitory action at different ISIs across all participants. Although the ISI × group interaction did not reach statistical significance (*F*_2,48_ = 3.131, *p* = 0.053), it approached significance.

### Sensory–motor integration

A mixed-model ANOVA for the SAI protocol revealed a significant main effect of ISI (*F*_1,48_ = 33.602, *p* < 0.001), indicating that the protocol inherently produced stronger inhibitory effects at shorter ISIs, consistent with physiological expectations. Additionally, there was a significant main effect of group (*F*_2,48_ = 4.475, *p* = 0.017), suggesting that SAI modulation differs across groups (Fig. 2F, G). Post hoc comparisons indicated that these differences were significant between healthy controls and TRS patients (*p* = 0.008) and between healthy controls and non-TRS patients (*p* = 0.033), while no significant difference was found between TRS and non-TRS patients (*p* = 0.609) (Fig. 2G). However, there was no significant ISI × group interaction (*F*_2,48_ = 0.521, *p* = 0.682), indicating that the observed group differences in SAI modulation are consistent across ISIs and do not depend on a specific ISI.

### Cortical plasticity induced by intermittent TBS

**Fig. 3:**
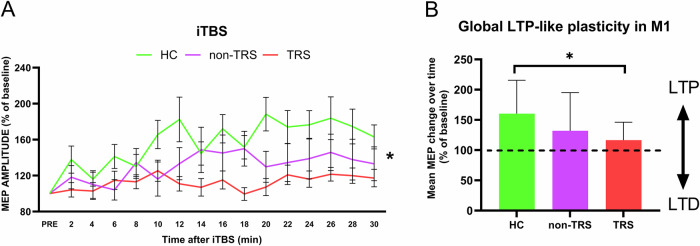
Long-term potentiation (LTP)-like plasticity in the primary motor cortex (M1) across TRS patients, non-TRS patients, and healthy controls. In (**A**), the time course of motor evoked potential (MEP) amplitude is shown, normalized to the pre-intermittent theta burst stimulation (iTBS) baseline. TRS patients exhibit a marked loss of LTP-like plasticity, as evidenced by reduced MEP amplitude changes over time compared to controls. The arrowheads indicate the timing of the iTBS intervention. **B** summarizes the overall MEP amplitude changes averaged over the 30-minute post-iTBS period. TRS patients show significantly diminished LTP-like plasticity compared to controls, while non-TRS patients display intermediate responses that fall between those of the other two groups. *Statistically significant for controls vs TRS (*p* < 0.05). LTP long-term potentiation, LTD long-term depression.

To assess corticospinal excitability following iTBS, we conducted a mixed-model ANOVA that revealed a significant main effect of group (*F*_2,44_ = 3.453 *p* = 0.040), indicating differential modulation of corticospinal excitability across groups. Post hoc analysis showed that this effect was significant between TRS and healthy controls (*p* = 0.008), indicating a decreased excitability enhancement in TRS patients compared to controls (Fig. 3A).

Furthermore, there was a significant effect of time (*F*_14,616_ = 3.319 *p* < 0.001), suggesting that iTBS-induced modulation of MEP amplitudes varies across time points, consistent with the expected temporal profile of corticospinal excitability increase. However, the time × group interaction did not reach statistical significance (*F*_28,616_ = 1.442; *p* = 0.130), indicating that the pattern of excitability changes over time was similar across groups.

Finally, to control for potential pharmacological confounding, we performed ANCOVA analyses on nine neurophysiological outcome measures, with group (TRS vs non-TRS) as a fixed factor and diazepam-equivalent and chlorpromazine-equivalent doses as covariates. The outcome measures included RMT, AMT, MEP1mV, mean SICI, mean ICF, EI, mean LICI, mean SAI, and mean LTP. After adjusting for the equivalent doses of psychotropic medications, no significant group differences were observed across the neurophysiological measures (all *p* > 0.085), with the exception of the EI, which remained significantly higher in TRS compared to non-TRS individuals (*F*_1,26_ = 4.96, *p* = 0.036). An overview of the ANCOVA results is provided in Supplementary Table 5.

### Psychopathological and cognitive correlates of neurophysiological abnormalities

We first investigated whether the neurophysiological alterations observed in subjects suffering from schizophrenia were associated with motor slowness in the dominant hand. Our analysis revealed a significant positive correlation between the motor slowness of the dominant hand, as assessed by the 9HPT, and the mean SICI (*r* = 0.389, *p* = 0.037). This suggests that altered inhibition is associated with greater motor slowness in the dominant hand, indicating that deficits in cortical inhibitory mechanisms may contribute to functional motor impairments in schizophrenia, particularly impacting the speed and dexterity of dominant hand movements.

To further assess the clinical relevance of cortical inhibitory alterations in schizophrenia, we performed correlation analyses between mean SICI and the PANSS total score, its subdomains, and specific symptom dimensions based on the five-factor PANSS model. After applying Bonferroni correction for multiple comparisons, significant correlations emerged, suggesting that increased cortical inhibition is associated with higher symptom severity across several domains.

**Fig. 4:**
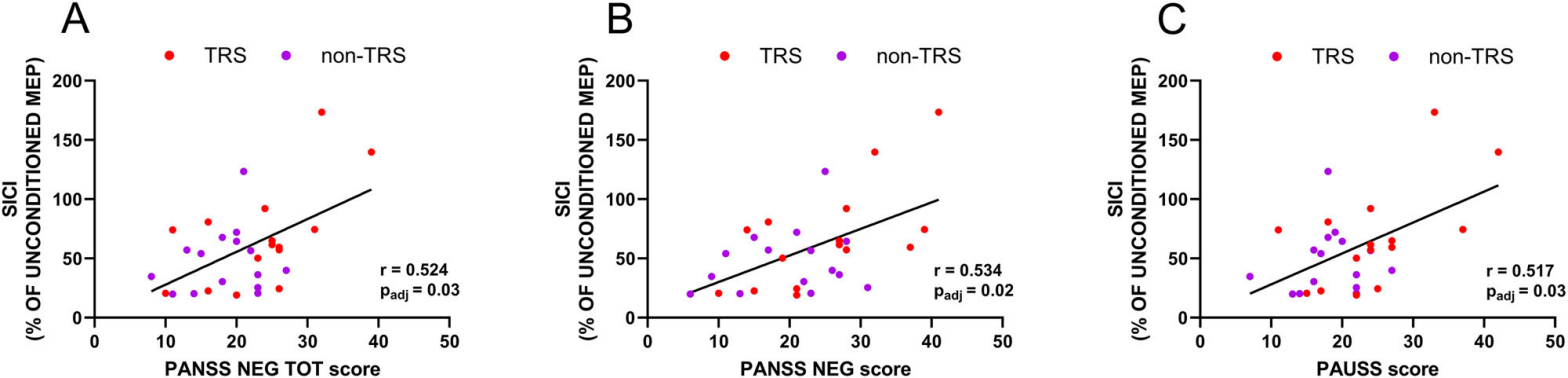
Clinical correlates of neurophysiological abnormalities in patients with schizophrenia. This figure illustrates the relationship between mean SICI, expressed as a percentage of unconditioned MEP, and clinical symptom severity in patients. **A** demonstrates a significant positive correlation between mean SICI and the PANSS Negative Total (PANSS NEG TOT) score (*r* = 0.524, *p*_adj_ = 0.03). **B** shows a significant positive correlation between mean SICI and the PANSS Negative Symptom (PANSS NEG) score calculated using the five-factor model (*r* = 0.534, *p*_adj_ = 0.02). **C** shows a significant positive correlation between mean SICI and the PAUSS score (*r* = 0.517, *p*_adj_ = 0.03). All correlations remain significant after adjustment for multiple comparisons (Bonferroni correction).

Significant positive correlations were observed between mean SICI and both the Negative Symptoms Total (*r* = 0.524, *p*_adj_ = 0.03) and Negative domain using the five-factor model (*r* = 0.534, *p*_adj_ = 0.02) (see Fig. 4). These findings indicate a strong relationship between altered cortical inhibition and the severity of negative symptoms in schizophrenia. Additionally, the mean SICI was significantly correlated with the PAUSS score (*r* = 0.517, *p*_adj_ = 0.03) (see Fig. 4), highlighting the association between inhibitory dysfunction and autistic symptoms. Although several other correlations did not reach the adjusted threshold for statistical significance, they were observed at the conventional significance threshold (Table 3). These findings suggest that cortical inhibitory dysfunction, as indicated by altered SICI, may be most strongly associated with negative symptoms, while other symptom dimensions exhibit weaker or non-significant relationships.

**Table 3:** Correlations between mean SICI and Positive and Negative Syndrome Scale.

Variable	Pearson’s test correlation coefficient	Spearman’s rank test correlation coefficient	*p* value	Bonferroni’s adjusted *p* value
PANSS Total		0.385	0.036	0.36
Positive Symptoms Total		0.322	0.082	0.82
Negative Symptoms Total	0.524		0.003	**0.03**
General Psychopathology Total	0.492		0.007	0.07
Positive Symptoms using the five-factor model		0.313	0.092	0.92
Negative Symptoms using the five-factor model	0.534		0.002	**0.02**
Disorganized Thinking using the five-factor model	0.417		0.022	0.22
Excited Symptoms using the five-factor model	0.479		0.007	0.07
Emotional Withdrawal using the five-factor model		0.301	0.106	1
Positive and Negative Syndrome Scale Autism Severity	0.517		0.003	**0.03**

Note: The correlation is analyzed using Pearson’s test and Spearman’s rank test, with significance assessed via unadjusted *p* values and Bonferroni-adjusted *p* values. Statistically significant values (after Bonferroni adjustment) are highlighted in bold.

## Discussion

Our study points to a progressive alteration in GABA-A-mediated inhibitory circuits and LTP-like cortical plasticity within M1 across different schizophrenia groups. TRS patients showed the most pronounced deficits, while non-TRS patients exhibited alterations that were intermediate between those observed in TRS patients and the profile seen in healthy controls. These findings could suggest a continuum model of neurophysiological dysfunction in schizophrenia, where the severity of inhibitory and plasticity-related deficits may be associated with the status of treatment resistance. This aligns with prior findings^41^, emphasizing the significant role of glutamatergic^5,6,42^ and GABAergic^10,11^ system abnormalities in TRS, alongside dopamine dysregulation.

Our study highlights SICI as a crucial parameter significantly altered in TRS, consistent with existing literature documenting GABAergic dysfunction indexed by SICI deficits across various stages of schizophrenia, from at-risk individuals to first-episode, recent-onset, and chronic patients^43^. These findings indicate that SICI alterations may represent a detectable neurophysiological deficit underlying schizophrenia, with the potential to serve as an early marker of vulnerability or predisposition to the disorder, as well as to the development of resistance.

Interestingly, only AMT—but not RMT—was significantly elevated in TRS patients. Motor thresholds probe the excitability of corticospinal circuits at different levels: RMT, set at higher stimulator intensities, recruits both the early I1-wave and later I-waves (I2, I3, etc.) generated by nested excitatory–inhibitory interneuronal loops, whereas AMT, measured during slight voluntary contraction and at lower intensities, primarily reflects the threshold for the pure, glutamatergic I1 volley^44^. Thus, a selective AMT increase could suggest a glutamatergic transmission impairment in TRS.

Several studies have indicated that TRS subjects are characterized by elevated intracortical levels of excitatory neurotransmitters or their metabolites^45^. Our findings align with the hypothesis that a dysregulation of both glutamatergic and GABAergic circuits, resulting in an excitatory/inhibitory imbalance, may disrupt cortical function in TRS. In line with this interpretation, we observed a progressive increase in EI ratio across groups- lowest in healthy controls, intermediate in non-TRS, and highest in TRS—suggesting a gradient of cortical disinhibition paralleling illness severity and treatment resistance.

Interestingly, our results do not reveal a significant relationship between SICI and antipsychotic medication dosage. A recent meta-analysis suggested that antipsychotics often contribute to restoring intracortical inhibition^16^. Although our groups did not differ significantly in chlorpromazine and diazepam equivalents, the TRS group, as expected given the treatment resistance, received higher overall therapy dosages. Notably, this finding is particularly striking considering that both benzodiazepines and antipsychotics can mitigate deficits in intracortical inhibition. Despite these pharmacological effects, the TRS group still exhibited significantly lower inhibition during SICI, underscoring the persistent nature of the inhibitory dysfunction in TRS. Therefore, it is conceivable that the inhibitory deficit observed in our TRS cohort may be even more pronounced than in non-TRS patients and healthy controls.

Following the observations of Lefebvre^46^, who identified a link between reduced cortical inhibition in schizophrenia and psychomotor slowing, we tested whether motor dexterity correlated with measures of intracortical inhibition, as assessed by SICI. Our results confirmed a significant relationship between reduced intracortical inhibition and impaired hand dexterity in TRS patients. Our findings suggest that deficits in cortical inhibition may play a critical role in motor dysfunction, possibly mediated by the presence of neural noise disrupting the precision and efficiency of motor signal processing, ultimately impairing motor coordination and execution. Moreover, in our study, we observed a significant relationship between SICI and both negative symptoms, as previously demonstrated^17^, and autistic traits. Thus, impaired SICI may underlie a broader disruption of neural circuits involved in emotional regulation, social behavior, and executive functioning, reflecting deficit symptoms and motor retardation.

Since SICI is associated with the GABA-A receptor pathway, while LICI reflects GABA-B receptor activity, the statistical significance of SICI alteration over LICI may suggest that the observed GABAergic dysfunction could be primarily linked to deficits in GABA-A receptor-mediated inhibition^47–49^.

In addition to impaired GABA-A-mediated intracortical inhibition (SICI), TRS patients exhibited diminished SAI. Although SAI is primarily considered a marker of cholinergic-dependent sensorimotor gating, there is evidence that GABA-A-mediated circuits also modulate this inhibition. Pharmacological TMS studies have shown that lorazepam—but not diazepam—markedly reduces SAI, despite both enhancing SICI via GABA-A receptors^37,50–52^. Indeed, the reduction in SAI might also be interpreted as a deficit in GABAergic inhibitory output mediated by basket interneurons targeting the perisomatic region of pyramidal neurons, which are modulated by cholinergic input from thalamocortical projections^53^. In TRS patients, the concurrent impairment of SICI and SAI may reflect a compounded deficit involving GABA-A-mediated inhibitory control. Nevertheless, a more complex interplay involving subcortical structures and primary cholinergic pathways cannot be excluded. Further, the simultaneous increase in AMT and reduction in SAI in TRS subjects therefore point to a dual hit on both glutamatergic drive and on inhibitory gating of incoming sensory inputs. When considering SAI alongside our EI finding—a net shift toward facilitation—and preserved RMT, this constellation suggests that TRS is characterized by a decoupling of excitatory and inhibitory processes: pure excitatory pathways (I1) become harder to recruit (higher AMT), while complex inhibitory networks fail to appropriately gate sensorimotor signals (lower SAI).

It should be noted that the present study is the first to evaluate LTP-like plasticity in schizophrenia using the iTBS protocol, revealing a significant reduction of plasticity in TRS patients compared to healthy controls. These findings align with previous evidence of impaired synaptic plasticity in schizophrenia^54–56^, pointing to a severe neuroplasticity deficit mediated by NMDA receptors in TRS patients. Notably, non-TRS patients displayed an intermediate plasticity response, suggesting a gradient of impairment within schizophrenia subgroups.

We recognize some limitations of the present study: first, our relatively small sample limits the generalizability of these findings; second, although benzodiazepine use was minimal, conversion into diazepam equivalents does not capture molecule-specific effects on neurophysiological measures including SAI, which are likely related to receptor-subtype selectivity^57^. Another important caveat in interpreting our findings is the potential impact of clozapine on the GABA/glutamate balance. Clozapine is known to affect both GABAergic and glutamatergic transmission, including increasing extracellular glutamate and modulating GABA release in specific brain regions^3^. Its peculiar pharmacodynamic profile may represent a major confound, attenuating potential differences between TRS and non-TRS patients^58–60^.

The heterogeneity of findings in the existing literature regarding TMS paradigms in schizophrenia, may be, at least in part, influenced by the variability of the excitability profile among patients, particularly when TRS and non-TRS subjects are grouped together. Future studies should prioritize stratification based on treatment resistance to better delineate the specific mechanisms underlying TRS and improve the consistency of research findings.

In conclusion, the present findings demonstrate that treatment resistance in schizophrenia is associated with marked impairments in GABA-A-mediated inhibition and cortical plasticity, suggesting a continuum of neurophysiological dysfunction that may reflect treatment responsiveness. These results highlight the relevance of exploring novel potential therapies targeting glutamatergic and GABAergic pathways as a therapeutic strategy for TRS.

## Supplementary information

Supplemental material

## Data Availability

Anonymized data may be shared upon request to the corresponding or senior author from a qualified investigator for non-commercial use, subject to restrictions according to participant consent and data protection legislation.
