# Supplementary material for: In vivo assessment of GABAergic inhibition and glutamate facilitation in treatment-resistant schizophrenia: a TMS study integrating clinical, cognitive, and neurophysiological evaluations
Source: Schizophrenia (Heidelb). 2025 Jun 19;11(1):90. doi: 10.1038/s41537-025-00634-w (PMC12179267; doi:10.1038/s41537-025-00634-w)
Supplement: Supplementary file 1 — Supplemental material [file 41537_2025_634_MOESM1_ESM.docx]

| **Inclusion Criteria** | **Exclusion Criteria** |
| --- | --- |
| Diagnosis of schizophrenia according to DSM-5, with no other concurrent or comorbid major psychiatric disorder. No current substance use disorder (excluding nicotine dependence). | History of epileptic seizures |
| Age between 18 and 65 years | Pregnancy |
| Consent to participate in the study | Severe neurological or cardiac conditions, or any major pathology that could interfere with the study results |
|  | Presence of medical devices that are incompatible with TMS (e.g., pacemakers, cochlear implants) |

**Supplementary Table 1. Inclusion and Exclusion Criteria for patients**

| **Inclusion Criteria** | **Exclusion Criteria** |
| --- | --- |
| No current or past diagnosis of psychiatric disorders assessed via clinical interview. No current substance use disorder (excluding nicotine dependence). | History of epileptic seizures |
| Age between 18 and 65 years | Pregnancy |
| Consent to participate in the study | Severe neurological or cardiac conditions, or any major pathology that could interfere with the study results |
|  | Presence of medical devices that are incompatible with TMS (e.g., pacemakers, cochlear implants) |

**Supplementary Table 2. Inclusion and Exclusion Criteria for healthy controls**

| **Group** | **Benzodiazepine**  **used** | **Number of patients** | **Daily dose range** | **Diazepam equivalent** | |
| --- | --- | --- | --- | --- | --- |
| Non-TRS | Flurazepam | 1 | 15 mg | 8 mg | Mean  3.23 mg |
|  | Lorazepam | 1 | 5 mg | 38 mg |  |
|  | Diazepam | 1 | 2.5 mg | 2.5 mg |  |
| TRS | Delorazepam | 4 | 0.20-2mg | 3.5 mg | Mean  7.7 mg |
|  | Diazepam | 1 | 10 | 10 mg |  |
|  | Lorazepam | 1 | 7.5 mg | 45 mg |  |
|  | Clonazepam | 2 | 1,3 mg | 20 mg |  |

**Supplementary Table 3.** **Benzodiazepine use in TRS and non-TRS patients at the time of neurophysiological assessment.**

| **Criteria for TRS Classification** | |
| --- | --- |
| Symptom persistence | Defined as a clinical response of less than 20% symptom reduction, measured using (e.g. Positive and negative syndrome scale) |
| Symptom severity | Severity of symptoms must be at least moderate |
| Number of antipsychotic trials | At least two trials with different antipsychotic medications, including at least one administered via Long acting injectable formulation |
| Antipsychotic dose | Chlorpromazine equivalents ≥600 mg/day |
| Treatment duration | Each trial must have lasted at least 6 weeks at therapeutic dose |
| Adherence | Considered adequate if the patient received at least the 80% of the prescribed dose |

**Supplementary Table 4. Treatment-resistant schizophrenia classification criteria according to Treatment Response and Resistance in Psychosis (TRIPP) working group consensus guidelines.**

| **Neurophysiological Variable** | ***F*** | ***P*** |
| --- | --- | --- |
| **RMT**  (% of MSO) | 0.178 | 0.677 |
| **AMT**  (% of MSO) | 2.166 | 0.153 |
| **MEP1mV**  (% of MSO) | 0.404 | 0.531 |
| **mean SICI**  (% of unconditioned MEP) | 1.845 | 0.186 |
| **mean ICF**  (% of unconditioned MEP) | 0.478 | 0.495 |
| **EI**  ICF/ (ICF − SICI) | 4.960 | **0.036** |
| **Mean LICI**  (% of unconditioned MEP) | 1.843 | 0.186 |
| **Mean SAI**  (% of unconditioned MEP) | 0.077 | 0.784 |
| **Mean LTP-like plasticity**  (% of unconditioned MEP) | 3.202 | 0.085 |

**Supplementary Table 5.** **Analysis of covariance (ANCOVA) comparing neurophysiological measures between treatment-resistant schizophrenia (TRS) and non-TRS patients, controlling for benzodiazepine (diazepam equivalent) and antipsychotic (chlorpromazine equivalent) dosages.**

Abbreviations: RMT, resting motor threshold; AMT, active motor threshold; MEP1mV, threshold to obtain 1mV amplitude MEP; I, mean inhibition; SICI, short-interval intracortical inhibition; E, mean facilitation; ICF, intracortical facilitation; EI, Excitation index; LICI, long-interval intracortical inhibition; SAI, short-latency afferent inhibition; LTP, long-term potentiation; MSO, maximum stimulator output. Significance threshold set at p < 0.05. Bold values denote statistically significant results.
